# Etiology of apneic episodes in infants aged <1 year and validation of brief resolved unexplained events risk-stratification criteria without age as a risk factor

**DOI:** 10.3389/fped.2026.1731324

**Published:** 2026-02-11

**Authors:** Sanna Hellström Schmidt, Jorge Sotoca Fernandez, Cornelis Jan Pronk, Ioannis Orfanos

**Affiliations:** 1 Department of Clinical Sciences Lund, Lund University, Lund, Sweden; 2 Department of Pediatrics, Skåne University Hospital, Lund, Sweden; 3 Childhood Cancer Center, Skåne University Hospital, Lund, Sweden; 4 Wallenberg Centre for Molecular Medicine and Division of Molecular Hematology, Lund University, Lund, Sweden

**Keywords:** apnea, brief resolved unexplained event, etiology, infant, risk stratification

## Abstract

The criteria for Brief Resolved Unexplained Events (BRUE) are used to stratify the risk in infants presenting with apneic episodes. However, these criteria often classify a high proportion of infants as higher risk. This study aimed to investigate the etiology of apneic episodes, particularly those compatible with BRUE, and to assess whether age ≤60 days is associated with serious underlying conditions. We conducted a retrospective study of infants aged <1 year who presented with apnea as the chief complaint or had an ICD-10 code P28.4 (Other apnea of newborn) recorded between 2014 and 2018 at Skåne Pediatric University Hospital. The cohort was divided into a BRUE subgroup, which was further stratified into lower- and higher-risk groups based on current BRUE criteria, and additionally stratified without considering age as a higher-risk factor. Infants with an ICD diagnosis related to respiratory tract infection (RTI) or clinical signs of RTI were categorized into a separate RTI sub-cohort. A total of 340 infants were included. No specific diagnosis was identified in 253 (74.4%) cases, while 63 (18.5%) were diagnosed with viral RTIs. There were 9 (2.6%) cases with serious conditions during the first encounter, all of whom appeared unwell or had alarming signs at presentation excluding them from a BRUE diagnosis. The BRUE sub-cohort included 188 infants, of whom 143 (76.1%) were classified as higher risk using current criteria. When age ≤60 days was excluded as a risk factor, only 45 (23.9%) were classified as higher risk. None of the infants with serious diagnoses were misclassified as lower risk under the modified criteria. The RTI sub-cohort included 158 infants, with 111 (70.3%) hospitalized; 90 of these (81.1%) were discharged within two days. Serious underlying conditions and complicated clinical courses were rare among well-appearing infants aged under 1 year, with apneic episodes. Age ≤60 days did not appear to be associated with serious diagnoses in the BRUE sub-cohort. Well-appearing infants with RTI symptoms also had benign outcomes. These findings suggest that age may warrant re-evaluation as a risk factor in BRUE stratification, and that well-appearing infants with RTI symptoms may be appropriately classified as low risk.

## 1 Introduction

Infants often present to the pediatric emergency department (PED) with apneic episodes characterized by cyanosis or pallor, changes in muscle tone (increased or decreased), absent, irregular, or decreased breathing, and alterations in mental status. The term apparent life-threatening event (ALTE) was introduced in 1988 to describe these events (1). Episodes of ALTE are often manifestations of normal infant physiology or benign self-limiting conditions, such as gastroesophageal reflux, viral infections, or breath-holding spells. Serious underlying disorders such as child abuse, congenital abnormalities, epilepsy, inborn errors of metabolism, or bacterial infections, are rare (2). Despite their often benign nature, these episodes typically cause significant stress to parents and pose management challenges for physicians, resulting in a high rate of hospitalizations and investigations and excessive costs (2–4).

To address this uncertainty, Tieder et al. suggested the term brief resolved unexplained event (BRUE), instead of ALTE and a set of criteria to identify infants with a lower risk of a serious underlying disorder that can be managed safely without diagnostic evaluations or hospitalization (5). For BRUE with higher risk, Merritt et al. proposed a framework to guide the clinical evaluation and management (6).

However, BRUE risk-stratification criteria are based on weak evidence because they were retrospectively derived from older and heterogeneous ALTE studies (2, 7, 8). Particularly age has not been shown to be associated with serious underlying conditions in infants with BRUE and results in ALTE studies are inconclusive (7–10). Thus, up to 87%–94% of infants with BRUE are still considered at a higher risk (8–10). However, serious underlying disorders are very rare, even in higher-risk BRUE infants (8, 10, 11). Since, there are no clinical practice guidelines for higher-risk infants, these infants often undergo excessive investigations and hospitalizations with low diagnostic yield (10–12). Investigations and hospitalizations can be harmful and stressful for infants, heighten parental anxiety, strain already overwhelmed emergency departments, and impose significant costs on the healthcare system (13, 14). Furthermore, infants with signs or symptoms of respiratory tract infection (RTI) are excluded from the BRUE definition. Most studies on ALTE/BRUE have been conducted in the United States and there is a paucity of data from other settings with different population characteristics. It is crucial with research from several countries to better understand the etiology of episodes of cyanosis or pallor, changes in muscle tone (increased or decreased), absent, irregular, or decreased breathing, and alterations in mental status in infants, as well as to validate the BRUE risk-stratification criteria.

This study aims to: 1) investigate the underlying etiology of apneic episodes in infants aged under 1 year and 2) assess whether age is associated with underlying conditions in a sub-cohort of infants meeting the diagnostic criteria for BRUE.

## 2 Materials and methods

### 2.1 Setting

The study was conducted at Skåne Pediatric University Hospital, a tertiary care center in southern Sweden serving primarily urban and suburban populations and providing universal healthcare, similar to other Nordic and many European countries. The hospital has two PEDs, one in Malmö and one in Lund. The study period was from January 1, 2014, to December 31, 2018. This study was approved by the Regional Ethics Committee of Lund, Sweden (Dnr 2017/967).

### 2.2 Study population

There are no available codes for BRUE or ALTE in the Swedish version of the International Statistical Classification of Diseases and Related Health Problems 10th Revision (ICD-10). Apnea has been a common clinical term and diagnosis code to describe apneic episodes characterized by cyanosis or pallor, changes in muscle tone, absent, irregular, or decreased breathing, and alterations in mental status. Thus, to identify infants aged <1 year with episodes which could be compatible with BRUE we retrospectively identified all infants under 1 year of age with: 1) “apnea” registered as chief complaint by the triage nurse in the PEDs' patient electronic registration systems or 2) with ICD-10 code P284 (Other apnea in newborn) registered as diagnosis. In the PEDs' electronic registration system, only one alternative can be selected from the drop-down menu. Except for seizures, there are no other alternatives that can register such episodes in the electronic registration system of PEDs. Infants identified by both methods were registered only once.

The infants' electronic medical records were reviewed, and infants with insufficient information on their initial visits were not considered eligible for the study. We included all infants with apneic episodes characterized by cyanosis or pallor, changes in muscle tone (increased or decreased), absent, irregular, or decreased breathing, and alterations in mental status.

We collected data on the infants' characteristics, previous chronic diseases, symptoms, investigation results, and initial diagnosis at the first visit. We also evaluated the subsequent visits. All diagnoses after the first visit or hospitalization, as well as any new chronic diagnoses after any subsequent visit, were registered regardless of their association with the symptoms at the initial visit. Data collection commenced in September 2019, ensuring that at least nine months had passed since the eligible emergency visit or diagnosis. Thus, the follow-up period was almost six years for the first included child and nine months for the last. The Data were entered into the Research Electronic Data Capture (REDCap) program hosted by Lund University, Sweden (15). Descriptive statistical analyses were conducted using Stata BE version 18.0.

### 2.3 Study definitions and analyses

Apneic episodes were defined as any events involving cyanosis or pallor, changes in muscle tone (increased or decreased), absent, irregular, or decreased breathing, and alterations in mental status.

Serious conditions were defined as: (1) any chronic disease (neurological, inborn error of metabolism, respiratory), (2) infections requiring treatment or supportive care, (3) admission to intensive care unit, or (4) trauma or child abuse.

To create a BRUE sub-cohort, we applied the diagnostic criteria described by Tieder et al. and Merritt et al. (5, 6), with the exception of repeated events and abnormal vital signs, for which data were unavailable (Table 1). Infants were excluded from the BRUE sub-cohort if they had an RTI-related ICD diagnosis or signs suggestive of RTI at the physical examination, such as runny nose, sneezing, or cough. These infants were categorized into a separate RTI sub-cohort. Infants with reported symptoms suggestive of RTI, such as runny nose, sneezing, or cough within the past five days but without observed signs or a RTI diagnosis were included in the BRUE sub-cohort and the RTI sub-cohort.

**Table 1:** Infants excluded from the BRUE sub-cohort and infants that fulfilled the higher-risk BRUE criteria.

Criteria for exclusion from BRUE sub-cohort^a^	Number of infants (%)^b^
Duration of apnea longer than 1 min	38 (11.2)
Fever in the last day	11 (3.2)
Hypo- or hypertonia	11 (3.2)
Not well or irritable	20 (5.9)
RTI symptoms at examination	84 (24.7)
Specific diagnosis^c^	87 (25.6)
Total infants excluded	152 (44.7%)
Higher-risk BRUE criteria^a^	Number of infants (%)^b^
Gestational age <32 weeks	1 (0.5)
Age (at symptoms) ≤60 days	132 (70.2)
Chronic disease	4 (2.1)
CPR by medical professional	0
Cough, sneezing or runny nose last five days^d^	40 (21.3)
Total higher-risk infants	143 (76.1%)
Total lower-risk infants^a^	45 (23.9%)

^a^ From Tieder et al. (5) and Merritt et al. (6).

^b^ Infants could fulfill more than one criterion, thus the total number is lower than the sum.

^c^ After pediatric emergency department visit or hospitalization.

^d^ Reported by parents but not noted in the medical files after the physical examination and not diagnosed with a RTI.

BRUE, brief resolved unexplained event; RTI, respiratory tract infection; CPR, Cardiopulmonary Resuscitation.

To further stratify the BRUE sub-cohort into lower- and higher-risk groups, we used the risk criteria outlined by Tieder et al. (5). Family history and postconceptual age ≥45 weeks as risk factors were not always available and therefore excluded from the stratification. Infants meeting any of the remaining high-risk criteria were classified as higher risk (Table 1).

**Figure 1:**
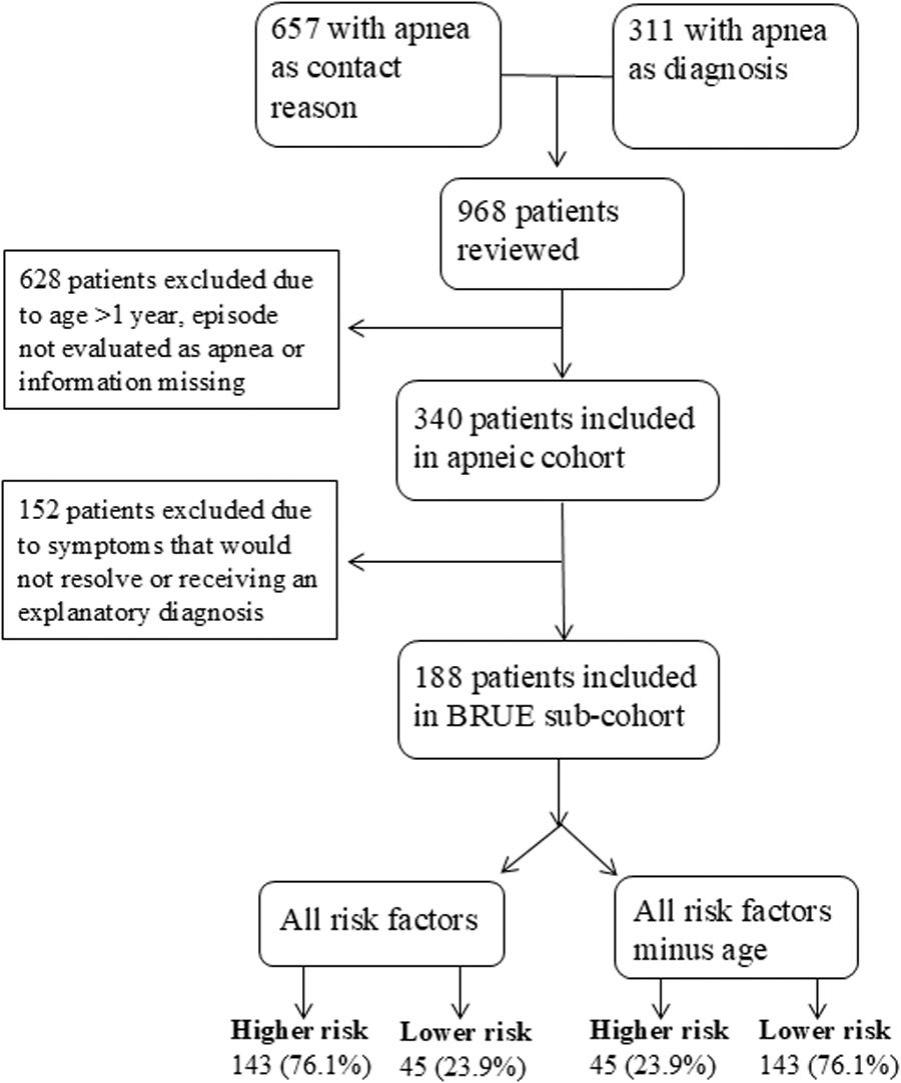
Inclusion of patients into the apneic cohort and further division into a BRUE sub-cohort and the BRUE risk-groups. Created in Powerpoint.

We also performed an alternative risk stratification within the BRUE sub-cohort, excluding age ≤60 days as a risk factor. This yielded two higher-risk groups: (1) infants meeting all available high-risk criteria (“all high-risk factors”), and (2) infants meeting all criteria except age ≤60 days (“all high-risk factors minus age”) (Figure 1).

### 2.4 Outcome measures

The primary outcome measures included 1) mortality or any diagnosis after the initial visit or admission (“first diagnosis”), 2) mortality or diagnosis of any chronic condition at any subsequent visit from the time of the eligible visit until the time of data collection (“subsequent chronic diagnosis”), and 3) revisits or re-admissions after the eligible visit.

## 3 Results

### 3.1 Study population

We identified 968 infants who presented with apnea or who had an apnea related ICD code. Of these, 628 were excluded because they were not assessed as having an episode of cyanosis or pallor, changes in muscle tone (increased or decreased), absent, irregular, or decreased breathing, and alterations in mental status, had incomplete medical records, or were older than 365 days of age (Figure 1). The final cohort included 340 infants, of which 171 (50.3%) females. The median age at the first visit was 38 (IQR 17–79.5) days, 228 (67.1%) were ≤60 days old, and 11 (3.2%) were born before 32 weeks of gestation. Twenty infants (5.9%) had preexisting chronic diseases. No deaths occurred during the follow up period. Median follow up time was 3.0 (IQR 1.9–4.4) years.

### 3.2 First diagnosis and subsequent chronic diagnoses

Of the 340 infants, a specific diagnosis was not established in 253 (74.4%). Among the 87 infants with specific initial diagnoses, viral respiratory tract infection was the most common diagnosed in 63 (18.5%), followed by gastroesophageal reflux (GER) in 10 (2.9%). There were 9 (2.6%) cases with serious conditions during the first encounter. These included 1 patient with enteroviral meningitis, 1 with pneumonia (positive for metapneumovirus at viral nasopharyngeal test), 1 coagulase-negative staphylococcal sepsis admitted to the neonatal intensive care unit, and 1 with a cytomegalovirus infection. Three patients were diagnosed with a seizure disorder, however 1 of these diagnoses was withdrawn after 1.5 years (Table 2). Additionally, there were 2 infants that were admitted to the pediatric intensive care unit: one diagnosed with a RTI (influenza) and one without a specific diagnosis. Both infants were unwell at the initial presentation.

**Table 2:** Diagnosis after the first visit or hospitalization of infants aged <1 year with apnea.

Diagnoses/Risk	Whole cohort
No diagnosis^a^	253
RTI^a^	63
GER	10
Benign diagnoses^b^	4
Serious infections^c^	4
Neurological	3
Pertussis	2
Congenital Lung Dysplasia	1
Total	340

^a^ One patient without specific diagnosis and one with viral RTI were admitted to the pediatric intensive care unit.

^b^ Benign diagnoses including minor infections and apnea-related specific diagnoses.

^c^ Enteroviral meningitis, pneumonia (metapneumovirus positive), cytomegalovirus infection, coagulase-negative staphylococcal sepsis/.

RTI, Respiratory Tract Infection; GER, Gastroesophageal Reflux.

Thirteen (3.8%) out of the 340 infants were diagnosed with a previously unknown chronic disease: 6 with neurological diagnoses (5 epilepsy diagnoses and 1 patient with delayed motor development, fractures and vitamin deficiency), 2 with GER, 1 with child abuse, 1 with gastritis, 1 with 18q-deletions syndrome, 1 with juvenile oligoarthritis, and 1 with osteogenesis imperfecta. No infants died during the initial study period or follow up. The follow-up time varied from 9 months to nearly six years, with median 3.0 (IQR 1.9–4.4) years from the initial visit to data extraction.

### 3.3 BRUE Sub-cohort and risk stratification

Among the 340 infants younger than 1 year with apneic episodes, 188 met BRUE diagnostic criteria (Table 1), of which 98 (52.1%) were females. The median age at the first visit was 32 (IQR 16–70.5) days, 132 (70.2%) were ≤60 days old, and 1 (0.5%) was born before 32 weeks of gestation. Four infants (2.1%) had preexisting chronic diseases. With the available high-risk BRUE criteria (Table 1), 143/188 (76.1%) infants were stratified as higher risk and 45/188 (23.9%) as lower risk. For 98 infants age was the only higher risk-factor, thus by excluding age as a risk factor, 45/188 (23.9%) were higher risk and 143/188 (76.1%) were lower risk.

All the 9 previously mentioned infants with serious conditions during the first encounter had clinical characteristics and/or received an explanatory diagnosis that excluded them from a BRUE diagnosis and the exclusion of age as a risk factor did not alter their stratification.

Three (1.6%) infants from the BRUE sub-cohort were diagnosed with a previously unknown chronic disease within nine months to nearly six years of their initial visit. All 3 infants were higher risk when all risk factors were considered, and 2 (one with an epilepsy diagnosis three years after the event and one case of child abuse) of them would have been stratified as lower risk if age was not a risk factor.

### 3.4 Hospitalization and revisits

Of the whole cohort, 203 (59.7%) infants were hospitalized after the first PEDs visit of which 166/203 (81.8%) were admitted for ≤2 days (Table 3). Eleven (3.2%) infants had a planned revisit within 10 days, while 17 (5.0%) had an unplanned revisit. These visits led to 5 readmissions. None of the revisits led to a new chronic diagnosis.

**Table 3:** Comparison of outcomes between the whole cohort, the BRUE sub-cohort, the two higher-risk groups, and the RTI sub-cohort.

Variables	Whole cohort	BRUE sub-cohort	Higher-risk BRUE^a^	Higher-risk BRUE^a^ age not a risk factor	RTI sub-cohort^b^
Total	340	188	143	45	158
New chronic diagnosis *n* (%)	13 (3.8)	3 (1.6)	3 (2.1)	1 (2.2)	6 (3.8)
Admission *n* (%)	203 (59.7)	95 (50.5)	84 (58.7)	34 (75.6)	111 (70.3)
Admission days, median (IQR, max)	1 (1–2, 15)	1 (1–1, 6)	1 (1–2, 6)	1 (1–2, 6)	1 (1–2, 15)
Admission ≤2 days *n* (%)^c^	166 (81.8%)	87 (91.6%)	77 (91.7%)	32 (94.1%)	90 (81.1%)
Revisits total *n* (%)	28 (8.2)	18 (9.6)	11 (7.7)	3 (6.7)	11 (7.0)
Readmissions total *n* (%)	5 (1.5)	3 (1.6)	2 (1.4)	1 (2.2)	2 (1.3)
Revisits unplanned *n* (%)	17 (5.0)	10 (5.3)	6 (4.2)	1 (2.2)	7 (4.4)
Readmissions unplanned *n* (%)	4 (1.2)	2 (1.1)	1 (0.7)	0	1 (0.6)

^a^ From Tieder et al. (5) and Merritt et al. (6).

^b^ Children with a history of RTI within the last 5 days, signs of a RTI at the visit, and those diagnosed with a RTI.

^c^ Percentage of patients with short admissions among the total amount of admission in the group.

BRUE, brief resolved unexplained event; RTI, respiratory tract infection.

In the BRUE sub-cohort, 95 (50.5%) infants were hospitalized after the first PEDs visit, of which 87 (91.6%) were admitted for ≤2 days (Table 3). Of the 8 infants admitted three or more days, 7 were considered higher risk with all BRUE criteria, while 5 of these 7 would be re-classified as lower risk if age was not considered a risk factor. However, in all these 5 cases the investigations during admission were without any pathological finding.

The RTI sub-cohort consisted of 158 infants who had a history of RTI symptoms, RTI symptoms on physical examination in the PED or received a viral RTI diagnosis after the first visit or hospitalization. Of these, 40 infants had only a history of RTI symptoms and did not exhibit RTI symptoms at the PED, and consequently they were not considered to have an RTI by the treating physician. These 40 children were nevertheless included in the BRUE cohort as higher risk. Overall 111 (70.3%) infants in the RTI sub-cohort were hospitalized after the first PEDs visit, of which 90 (81.1%) were admitted for ≤2 days (Table 3).

## 4 Discussion

In this retrospective study, we investigated the underlying etiology of episodes of cyanosis or pallor, changes in muscle tone, absent, irregular, or decreased breathing, and alterations in mental status in infants aged <1 year in two PEDs in southern Sweden. We also investigated whether age was associated with serious underlying conditions. We identified very low rates of serious underlying conditions and that age was not associated with higher risk of serious underlying disease when present as the only higher-risk factor in the BRUE sub-cohort. Thus, we believe that age should be re-evaluated as a higher-risk criteria in BRUE risk-stratification.

In our study, a specific diagnosis was not established in 74.4% of the infants, and benign conditions such as viral respiratory infections or GER were the most common identifiable causes. Similar results were reported in previous studies, with no diagnosis (50%) and GER being the most common non-serious diagnoses (8, 10). Other studies have reported a lower proportion (11%–36%) of infants without a specific diagnosis (2, 11). A serious illness or a complicated course of care was identified in only a few infants in the whole cohort and in none in the BRUE sub-cohort. The prevalence of serious underlying diagnoses has been reported to be 4%–9% in previous BRUE studies (8, 10, 11). In addition, 13 (3.8%) infants were diagnosed with a chronic disease at a subsequent visit, with epilepsy being the most common and 1 case of child abuse. But, in only 5 (i.e., 3 cases of epilepsy, the case of child abuse and the case of chromosomal deletion) of these 13 infants the apneic episode at the index visit may be considered associated to the diagnosis. However, it is uncelar whether an earlier diagnosis was feasible or whether an earlier diagnosis would have altered the prognosis. Only in the case of child abuse, might an earler detection have altered the outcome, but child abuse is a very rare cause of apneic episodes and it is often difficult to detect, especially at the first encounter (7, 16). Thus, the findings of our study provide evidence that serious etiologies are rare in well-appearing infants with episodes of altered breathing.

In our study, 76.1% of infants were stratified as higher risk according to the available BRUE risk-stratification criteria. This finding is in line with most BRUE studies in which up to 94% of infants were stratified as higher risk (10). By removing age as a risk factor, the percentage of higher- and lower-risk patients is reversed. The number of infants with new chronic diagnoses was low and none of these infants would have been misdiagnosed as lower risk, except the case of child abuse. Similar findings have been reported in previous BRUE studies, where age was not associated with serious underlying conditions (8–10). Thus, the findings of this study do not support age as risk factor for a serious underlying disease. Removing this risk factor would significantly reduce the proportion of infants classified as higher risk and consequently reduce hospitalizations and investigations. Hospitalizations and investigations cause discomfort for infants and stress and financial burdens for parents. They increase the work load on already overextended emergency departments and inpatient wards. Also, they result in substantial economic expenses for the healthcare system (13, 14). We believe that reducing low-value care, focusing on sustainability, and mitigating the environmental impact of the health care system should gain more focus when developing management guidelines.

A large percentage of the infants with episodes of apnea had either a history or symptoms of RTI. Admission-rates were high among the infants with RTI, but most admissions lasted less than 2 days. So short duration is indicative of favorable outcome, low need for interventions (i.e., respiratory support), and of investigations, if performed, without pathological findings. Thus, our data suggest that well-appearing infants with apneic episodes and RTI symptoms should not be routinely regarded as high risk, admitted to the hospital, and undergo any investigations if there are not specific clinical indications.

### 4.1 Limitations and strengths

Our study has some limitations. First is the identification method. Due to the lack of a specific BRUE diagnose code in the Swedish ICD-10 version, we used apnea as ICD-10 diagnosis or contact reason to identify infants with episodes compatible with BRUE. It is likely that such episodes (i.e., episodes of cyanosis or pallor, changes in muscle tone, absent, irregular, or decreased breathing, and alterations in mental status) could have been coded with another ICD-10 diagnosis, especially in infants with a specific diagnosis. However, using a second identification method (i.e., contact reason), we believe that we have reduced the likelihood of missing a significant number of eligible patients. However, by limiting inclusion to patients with apneic events we introduced a selection bias towards apneic BRUE.

Second, the duration of the episodes of changed breathing was not considered, which resulted in inclusion of infants with a duration of the episodes of less than 20 s.

Third, the lack of all required data for BRUE risk-stratification probably resulted in the misclassification of higher-risk infants as lower risk. However, the low rates of serious underlying diseases and complicated clinical courses strengthen our hypothesis that infants with BRUE are over-classified as having a higher risk.

## 5 Conclusions

Serious underlying diseases were very rare in well-appearing infants aged <1 year with episodes of cyanosis or pallor, changes in muscle tone, absent, irregular, or decreased breathing, and alterations in mental status. Age did not appear to be associated with serious underlying diagnoses in a sub-cohort of infants fulfilling the BRUE diagnostic criteria. Thus, we believe that age may warrant re-evaluation as a risk factor in the BRUE risk-stratification criteria. Also, well-appearing infants with RTI still had favorable outcomes, thus they should not be routinely regarded as high risk and admitted to the hospital if there are not specific clinical indications.

## Data Availability

The datasets presented in this article are not readily available because of protection of study participant privacy. Data can be shared upon reasonable request to the corresponding author. Requests to access the datasets should be directed to sanna.hellstrom_schmidt@med.lu.se.
